# The role of achievement attribution in the associations between parent–child communication and psychological well-being among adolescents: A mediation analysis

**DOI:** 10.1192/j.eurpsy.2022.2314

**Published:** 2022-08-31

**Authors:** Ningning Li, Yuting Li, Xinxin Huang, Siying Xiang, Qianying Hu, Chao Luo, Peijun Ju, David Mellor, Yifeng Xu, Hui Fei, Jianhua Chen

**Affiliations:** 1 Shanghai Mental Health Center, Shanghai Jiao Tong University School of Medicine, Shanghai Institute of Traditional Chinese Medicine for Mental Health, Shanghai Clinical Research Center for Mental Health, Shanghai Key Laboratory of Psychotic Disorders, Shanghai, China; 2 School of Psychology, Deakin University, Burwood, Victoria, Australia

**Keywords:** Achievement attribution, depression, parent–child communication, subjective interpersonal popularity, subjective well-being

## Abstract

**Background:**

Previous studies have explored the association between parenting style and offspring’s psychological well-being, and the association between offspring’s achievement attribution pattern and psychological well-being. However, little is known about the role of offspring’s achievement attribution in the relationship between parenting and offspring’s psychological well-being. We aimed to find the role of adolescents’ achievement attribution pattern in the relationship between parent–child communication quality and adolescents’ mental health.

**Methods:**

A cross-sectional analysis was conducted on 2,725 adolescents aged from 9 to 18 years who are participating in the China Family Panel Studies. Participants supplied demographic information and completed a series of psychological scales including the Center for Epidemiologic Studies Depression scale, an adapted version of the Parental Bonding Instrument, an achievement attribution scale, and single-item measures of subjective well-being and subjective interpersonal popularity.

**Results:**

Linear regression analysis revealed that after controlling for demographic factors good parent–child communication negatively correlated with depression symptoms, and positively associated with subjective well-being and subjective interpersonal popularity. Next, mediation analysis found that internal attribution of achievement partly mediated the effects of parent–child communication quality on adolescents’ depression, subjective well-being, and subjective interpersonal popularity. The result was robust after controlling demographic variables.

**Conclusions:**

An internal attribution pattern of achievement partially accounted for the associations between parent–child communication quality and adolescents’ psychological outcomes including depression, subjective well-being, and subjective interpersonal popularity. Future interventions for adolescents’ mental health promotion can target parent–child communication and adolescents’ positive achievement attribution pattern.

## Introduction

Adolescence is a stage of rapid psychological and physical development, during which individuals learn to develop their own unique outlooks and behaviors. However, 10–20% of children and adolescents worldwide are affected by mental health problems, which should be attended to and prioritized [1, 2].

Parenting style composed of parents’ attitudes and behaviors in daily interactions with offspring has been reported to influence children’s mental health [3]. Different researchers have identified different dimensions of parenting, such as two dimensions of care and overprotection, or three dimensions of emotional warmth, rejection, and protection [4, 5]. Eun et al. found that children who perceived high levels of maternal care were less likely to exhibit depressive symptoms, eating and behavioral disorders, while higher perceived paternal control was associated with greater odds of alcohol abuse. Ma et al. [6] reported that better daily interactions between parents and adolescents are a protective factor against depression in adolescents. Parenting style also influences children’s subjective well-being (SWB), defined as self-evaluation on how well life is according to the individuals’ own standards [5, 7, 8]. Few studies have explored the influence of parenting style on subjective interpersonal popularity (SIP) or the level of individuals’ satisfaction with interpersonal relationships, which correlates with negative emotions such as depression as well [9].

Another variable that may be related to adolescents’ mental health is the adolescents’ achievement attribution tendency. Achievement attribution refers to how individuals explain their own or others’ success. It influences the expectations of achievement and the adaption of future behaviors [10] as well as psychological outcomes such as depression, SWB, and SIP [11, 12]. One study has reported the mediation role of achievement attribution pattern in the influence of parenting style on adolescents’ academic outcomes. Adolescents perceiving nonauthoritative parenting style tended to ascribe achievements to external factors or low individual capacity, which led to lower school attendance [13]. However few studies have explored the role of achievement attribution pattern in the relationship between parenting style and children’s psychological outcomes.

In the current study, we aimed to first investigate the associations of one aspect of parenting, specifically, parent–child communication, with adolescents’ psychological outcomes including achievement attribution, depression symptoms, SWB, and SIP. Next, we aimed to explore the role of achievement attribution tendency in the relationships between parent–child communication and the remaining psychological outcomes in order to better understand the pathway between parent–child interactions and adolescents’ mental health and thereby inform interventions designed to promote adolescent mental health. We hypothesized that better parent–child communication will predict better psychological outcomes and that internal attribution (IA) would mediate the relationships between parent–child communication and the other psychological outcomes.

## Methods

### Data source and study sample

**Figure 1.**
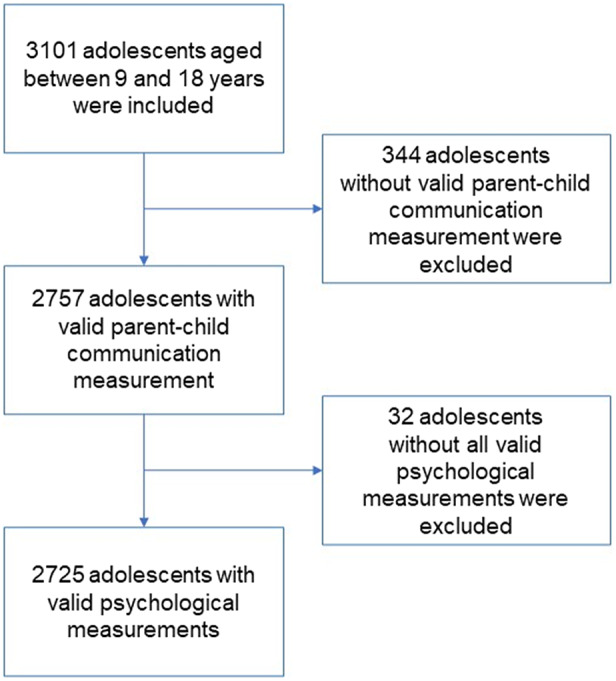
Flowchart of sample selection.

Data used for this study were obtained from the China Family Panel Studies (CFPS), a national longitudinal survey, carried out by the Institute of Social Science Survey (ISSS) at Peking University. CFPS covers 25 provinces (or municipalities) in China, representing 95% of the total population. To date CFPS has completed six rounds of surveys, beginning in the year 2010, and every 2 years since then. CFPS tracks and collects data at the individual, family, and community levels to reflect society, economy, demography, education, and health changes in China. All participants sign informed consent forms. The 2020 survey included a series of psychological scales including the Center for Epidemiologic Studies Depression scale (CES-D), an adapted version of the Parental Bonding Instrument (PBI), an achievement attribution scale, a well-being measure, and an interpersonal popularity measure. Of the 28,590 individuals who participated in this survey, there were 3,101 adolescents aged between 9 and 18 years. The average age was 13.71, with 1,390 female adolescents accounting for 47.5% of the total. Of these, 2,725 completed all the required measures and were included in our study. A detailed flowchart is shown in Figure 1 and the detailed demographic characteristics are shown in Table 1.

**Table 1.** Sample characteristics.

Baseline characteristics	Values
Characteristics of child	
Gender (Female, %)	1,302 (47.8)
Age (mean [SD])	13.62 (2.47)
Age level (%)	
9–12	1,033 (37.9)
13–15	978 (35.9)
16–18	714 (26.2)
Education year (mean [SD])	7.21 (2.55)
Education level (%)	
0–3 years	138 (5.1)
4–6 years	1,013 (37.2)
7–9 years	983 (36.1)
≥10 years	591 (21.7)
Exercise frequency (More than or equal to once a week, %)	1,488 (54.6)
PBI (mean [SD])	25.41 (4.78)
Achievement attribution scale	
External attribution (mean [SD])	6.17 (1.69)
Internal attribution (mean [SD])	8.21 (1.62)
CES-D8 (mean [SD])	4.35 (3.45)
Subjective well-being (mean [SD])	8.12 (1.89)
Subjective interpersonal popularity (mean [SD])	6.99 (1.92)
Characteristics of parents	
Maternal age (mean [SD])^a^	40.31 (5.78)
Maternal age level (%)^a^	
21–40	1,256 (54.2)
>40	1,063 (45.8)
Paternal age (mean [SD])^a^	42.27 (5.87)
Paternal age level (%)^a^	
28–40	939 (41.0)
>40	1,353 (59.0)
Education year of mother (mean [SD])^a^	7.55 (4.21)
Education level of mother (%)^a^	
0–3 years	399 (17.4)
4–6 years	466 (20.4)
7–9 years	996 (43.6)
≥10 years	426 (18.6)
Education year of father (mean [SD])^a^	8.66 (3.65)
Education level of father (%)^a^	
0–3 years	182 (8.1)
4–6 years	403 (17.9)
7–9 years	1,110 (49.2)
≥10 years	562 (24.9)
Employment of mother (Employed, %)^a^	1,700 (83.7)
Employment of father (Employed, %)^a^	1,851 (96.6)
Marriage state (Being married or living together, %)^a^	1,907 (96.6)
Characteristics of household	
Living in rural or urban areas (%)^a^	
Rural areas	1,531 (57.0)
Urban areas	1,153 (43.0)

Abbreviations: CES-D8, Center for Epidemiologic Studies Depression scale; PBI, parenting bonding instrument.

a There are 406 missing data points, 433 missing data points, 438 missing data points, 468 missing data points, 694 missing data points, 808 missing data points, 750 missing data points, and 41 missing data points for maternal age, paternal age, maternal education, paternal education, maternal employment, paternal employment, parental marriage state, and living areas, respectively.

### Exposure measure

The Parental Bonding Instrument (PBI) developed by Parker [4] was adapted by ISSS for the 2020 survey. Seven items assessing the adolescent’s perception of the communications pattern between parents and the adolescents were included. Participants were asked how often in the past 12 months when they did something wrong, their parents/guardians asked for reasons and guided them; encouraged them to do things with great effort; were gentile while talking with them; encouraged them to think independently; gave them reasons when asking them to do something; liked to talk with them; and praised them. Participants responded to these items on a scale ranging from one (never) to five (always), with two signifying rarely, three sometimes and four often. Scores also range from 1 to 5, and total score range from 7 to 35. Cronbach *α* of this seven-item scale was 0.82.

### Mediator measure

The achievement attribution measure adopted in this survey was a seven-item scale designed for the Chinese population [11]. Participants were asked about the importance of family’s social status, family’s economic condition, education level, gifted talent, effort, luck, and family social connections to future achievements. Answers to every item were scored from 0 to 10. The scale has two factors: external attribution (EA; family’s social status, family’s economic condition, gifted talent, luck, and family social connections) and IA (education level and effort). The Cronbach *α* of the EA and IA were 0.76 and 0.62, respectively.

### Outcome measure

The eight-item short version of the CES-D developed by Radloff [14] was adopted to evaluate the depressive symptoms. The CES-D has been widely used in large international investigations [15, 16], and the CES-D8 is a brief version that takes less time and is more acceptable to respondents [17, 18] who are asked to indicate how often in the past week they have felt depressed, felt everything that they did was an effort, slept restlessly, were happy, felt lonely, felt sad, enjoyed life, and could not get going. There are four response options: Hardly (less than a day); Sometimes (1–2 days); Often (3–4 days); and Most of the time (5–7 days). Responses are scored 0, 1, 2, or 3, respectively, with the two items indicating positive feelings reverse scored. The total CES-D8 score ranges from 0 to 24. Cronbach α for the eight-item scale was 0.77.

SWB was assessed by a single question “How happy are you”. Participants responded using an 11-point scale ranging from 0 (lowest score) to 10 (highest score). Likewise, participants’ SIP was assessed by a single question: “How popular are you with people?” with response options ranging from 0 (lowest score) to 10 (highest score).

### Covariates

Covariates at individual, parent, and household levels were recorded. Potential confounding factors at the individual level included age, gender, the degree of education, and frequency of exercise. Potential confounding factors at the parental level included age, education, marital status, and employment status. The potential confounding factor at the household level was whether the family lived in an urban or rural area.

### Statistical analysis

Firstly, we conducted descriptive analyses of the psychological and demographic characteristics of participants. Continuous variables and categorical variables are displayed as mean (standard deviation) and percentages, respectively, in Table 1. A series of linear regression analyses were used to explore the associations of parent–child communication with offspring’s achievement attribution, depression, SWB, and SIP. To test the robustness of this finding, we performed multiple linear regressions controlling for the demographic factors.

Next, we examined the mediation effect of achievement attribution tendency in the relationships between parent–child communication and offspring’s depression, SWB, and SIP. We conducted the Harman’s single-factor test first to examine the common method bias by SPSS 26.0 and obtained a variance of 24.49% for the first common factor, indicating that there was no common method bias. A structural equation model was evaluated using the R package “lavaan.” We packaged the items of CES-D8 into two parcels according to “internal-consistency approach” (one parcel of six items and one parcel of two items) and the items of PBI into three parcels according to “item-to-construct balance approach” (one parcel of three items and two parcels of two items) (Supplementary Material) [19]. The structure of the packaged CES-D8 and PBI items was tested by confirmatory factor analysis, and the packaged parcels were included in the measurement model of structural equation model. As for structure model, achievement attribution was set as the mediator, parent–child communication was the independent factor, and depression symptoms, SWB, and SIP were dependent variables. Further, we included demographic variables as control factors to test the robustness of the model. For all paths, standardized indirect and total effects were estimated. Bootstraping (1,000 replications) to test the significance of indirect and total effects was conducted.

Goodness of overall fit was evaluated as follows: *χ* ^2^/*df* < 3 [20], comparative fit index (CFI) > 0.95, Tucker-Lewis index (TLI) > 0.95 [21], adjusted goodness-of-fit index (AGFI) > 0.9 [22], and root mean square error of approximation (RMSEA) < 0.08 [23]. The value of *χ* ^2^ was influenced by sample size and the complexity of the model, so normed *χ* ^2^ (*χ* ^2^/*df*) was taken into consideration. The closer the values of CFI, TLI, and AGFI approach 1, the better the model fits. The closer the value of RMSEA approaches 0, the better the model fits.

## Results

### Sample characteristics

The study included 2,725 participants aged between 9 and 18 years. All participants had valid psychological measurements. Specific psychological measurements and demographic characteristics were all shown in Table 1.

### Associations between parent–child communication and adolescents’ psychological well-being

We hypothesized that parent–child communication would predict adolescent offspring’s psychological outcomes. The linear regression analyses showed parent–child communication was significantly associated with adolescent offspring’s IA (*β* = 0.152, *p* < 0.001), depression symptoms (*β* = −0.296, *p* < 0.001), SWB (*β* = 0.356, *p* < 0.001) and SIP (*β* = 0.141, *p* < 0.001), while it was not significantly associated with EA. To control for the influence of demographic variables, we included potential cofounding factors at adolescent level and at three levels of adolescent, parent, and household, respectively, the associations of parent–child communication still remained significant with offspring’s psychological outcomes except EA (see Table 2).

**Table 2.** Linear regression analyses of parenting-child communication on adolescents’ psychological outcomes.

	Model 1	Model 2	Model 3	Adj. *R* ^2^ (model 1)	Adj. *R* ^2^ (model 2)	Adj. *R* ^2^ (model 3)
EA	0.000	0.002	0.028	/	/	/
	(0.019)	(0.019)	(0.022)			
IA	0.152*	0.148*	0.173*	0.023	0.027	0.032
	(0.019)	(0.019)	(0.022)			
CES-D8	−0.296*	−0.293*	−0.283*	0.087	0.087	0.085
	(0.018)	(0.018)	(0.021)			
SWB	0.356*	0.355*	0.358*	0.127	0.142	0.142
	(0.018)	(0.018)	(0.020)			
SIP	0.141*	0.138*	0.169*	0.019	0.025	0.041
	(0.019)	(0.019)	(0.022)			

*Note:* Standardized regression coefficients are displayed, with standard errors in parentheses. Model 1, raw model; model 2, including potential cofounding factors at adolescent level (gender, education level, exercise frequency); model 3, including potential cofounding factors at parental and household levels in addition to model 2 (parental age, parental education, and living areas).

Abbreviations: CES-D8, Center for Epidemiologic Studies Depression scale; EA, external attribution; IA, internal attribution; PBI, parenting bonding instrument; SIP, subjective interpersonal popularity; SWB, subjective well-being.

* *p* < 0.001.

### The mediating effect of achievement attribution tendency

**Figure 2.**
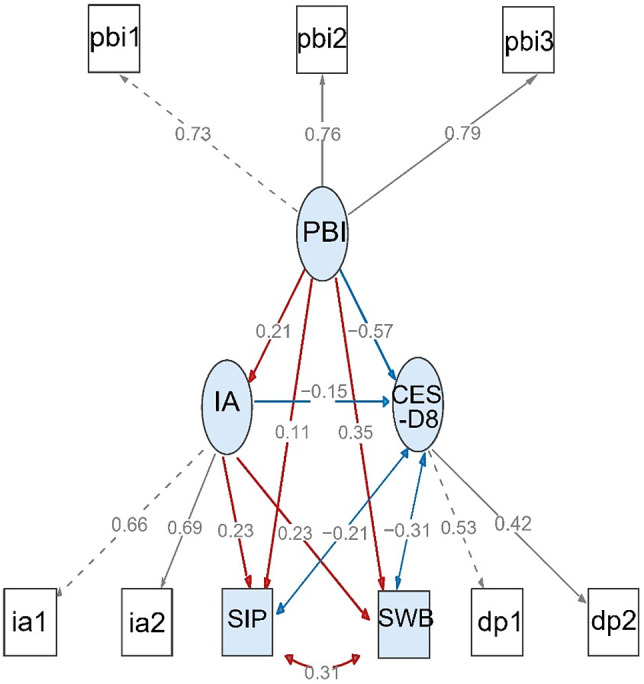
Mediation analysis of achievement attribution pattern in the associations between parent–child communication and adolescents’ depression symptom, subjective well-being, and subjective interpersonal popularity. The overall structural equation model has two parts, the measurement model (white nodes and connected edges) and the structural model (blue nodes and connected edges). In the measurement model, factor loadings of nodes connected by dotted lines are fixed at one and are presented as standardized form. In the structure model, probable causation relationship is shown with a single arrow, correlation with a double arrow. Red arrows represent positive effects and blue negative effects. Numerals stand for standardized coefficients. CES-D8, Center for Epidemiologic Studies Depression scale; IA, internal attribution; PBI, Parenting Bonding Instrument; SIP, subjective interpersonal popularity; SWB, subjective well-being.

We hypothesized that IA, one of the factors of achievement attribution would mediate the relationships between parent–child communication and the other psychological outcomes. Figure 2 shows the detailed overall model. Indicators of model fit are as follows: *χ* ^2^ = 21.937, *p* = 0.287, *χ* ^2^/*df* = 1.155; CFI = 0.999; TLI = 0.999; AGFI = 0.996; RMSEA = 0.008, (90% CI, 0.000, 0.019), indicating a good model fit. As shown in Figure 2, white nodes and connected edges represent the measurement model and numerals on connecting lines represent standardized factor loads. The factor loadings of CES-D8, PBI, and IA range from 0.42 to 0.79 all with significance. Blue nodes and connected edges compose the structural model. One-way arrows represent probable causality, and bipolar arrows represent correlation. Red and blue arrows represent positive effects and negative effects, respectively. Numerals on connecting edges represent standardized coefficients ranging from −0.57 to 0.35 all with statistical significance.

The indirect effects through IA tendency of achievement and total effects of parent–child communication on adolescents’ psychological outcomes are shown in Table 3. As can be seen, IA tendency partly mediates the relationships between parent–child communication and adolescents’ psychological outcomes. For depression symptoms, IA mediates 5.3% of the effects indirectly, for SWB and SIP, IA mediates 12.3 and 31.8%, respectively. The overall model including demographic variables as control factors was tested and also showed a good fit as well (Supplementary Table S1).

**Table 3.** The total effects of parent–child communication and the indirect effects through achievement attribution on adolescents’ psychological well-being.

	Estimate	S.E.	*z*	*p*		LLCI	ULCI	Beta
Defined effects								
*Depression symptoms*								
Indirect effect	−0.018	(0.006)	−3.129	0.002	*	−0.030	−0.007	−0.032
Total effect	−0.353	(0.025)	−14.068	<0.001	†	−0.406	−0.306	−0.606
*Subjective well-being*								
Indirect effect	0.146	(0.025)	5.751	<0.001	†	0.098	0.199	0.049
Total effect	1.179	(0.071)	16.703	<0.001	†	1.039	1.321	0.398
*Subjective interpersonal popularity*								
Indirect effect	0.150	(0.027)	5.635	<0.001	†	0.099	0.204	0.050
Total effect	0.473	(0.066)	7.145	<0.001	†	0.342	0.599	0.157

*Note:* Beta, the standardized estimates; “LLCI”, the lower limit of 95% confidence interval of coefficients; “ULCI”, the upper limit of 95% confidence interval of coefficients.

* *p* < 0.01;

† *p* < 0.001.

## Discussion

As hypothesized, we found moderate associations between the quality of day-to-day parent–child communication and adolescents’ psychological outcomes. Parent–child communication accounted for 8.5% of the variance in adolescent depression, consistent with previous studies [24]. The proportion of variance explained by parent–child communication in adolescents’ SWB was a little higher, 14.2%, but the proportion for SIP was lower, 4.1%. Better parent–child communication, as perceived by adolescents, helps to reduce their depressive symptoms and to promote SWB and SIP.

Mediation analysis revealed that IA of achievement partially mediates the associations between parent–child communication and psychological well-being. Better parent–child communication helps adolescents ascribe their achievements to their own effort or growth through education. Consequently, a tendency to attribute achievement to effort or education engagement helps to reduce individuals’ depression symptoms, and improve SWB and SIP. These findings are consistent with previous attribution theories. Children’s achievement attribution pattern is usually affected by the attitudes and behaviors of significant others like parents or guardians [25]. McFarland and Ross [26] and Weiner [27] have suggested attribution styles were the determinants of mood reactions to success or failure, ascribing achievements to internal factors could produce more positive emotion and less negative emotion. Studies have linked parental factors, children’ attribution pattern, and academic outcomes, which suggested that parental attribution style interacted with children’s attribution style, and affected children’s academic achievements [28–30]. In the background of Chinese culture, previous researches revealed parents tended to ascribe children’s achievement to working hard and this pattern of attribution could predict children’s academic achievement [31], and children inclined to ascribe success or failure to internal factors like ability and effort than external factors [32]. The interactions between parents and adolescents and adolescents’ achievement attribution in our study showed this similar pattern. Further, we linked parent–child communication, adolescents’ achievement attribution pattern, and mental health, revealing the importance of parent–child communication like encouraging autonomy and effort in adolescents’ positive attribution and mental health. Better communication perceived by adolescents from their parents can bring encouragement, confidence, and warmth, and helps them to ascribe achievement to their internal factors like efforts in some degree, which facilitates a positive attribution pattern formation and improved psychological well-being. Notably, the outcome variables in the mediation analysis we conducted incorporated depression, SWB, and SIP, showing the mutual correlations of these mental health indicators, and reflecting mental health as a whole.

Till now few studies have connected parent–child communication, achievement attribution pattern, and psychological well-being together to explore their relationships. Our study provided insights into the associations of parent–child communication and achievement attribution pattern with adolescents’ mental health, which can inform future interventions for adolescents’ mental health promotion. While our sample size is relatively large which potentially makes our results robust, our study has some limitations. Firstly, our study utilized a cross-sectional design, and future longitudinal studies are needed to inform causality in the explored relationships. Secondly, the proportions of variance explained by parent–child communication quality in adolescents’ psychological outcomes were relatively small, which may indicate there were potential confounders or other important variables that were not measured. Although it is difficult to identify and include all possible confounders, the proportions of variance we explained are consistent with those reported in past studies. Thirdly, we experienced some missing data which may impacted the findings. Fourthly, the scales used were based on self-reports which may bring reporting bias, though our statistical test showing there existed no common method bias. Fifthly, the measurements of SWB and SIP were both based on a single item, which may bring potential bias. Sixthly, the measurement of parent–child communication did not distinguish between fathers and mothers, an issue which needs further exploration.

In conclusion, our research revealed that parent–child communication associates with adolescents’ psychological outcomes including depression symptoms, SWB, and SIP. These relationships are partly mediated by adolescents’ IA of achievement. Interventions targeting improved parent–child communication and positive achievement attribution formation may promote adolescents’ mental health.

## Data Availability

Data were available from http://www.isss.pku.edu.cn/cfps/sjzx/gksj/index.htm.

## Abbreviations

CES-D: Center for Epidemiologic Studies Depression
EA: external attribution
IA: internal attribution
PBI: parenting bonding instrument
SIP: subjective interpersonal popularity
SWB: subjective well-being

## Supplementary material

For supplementary material accompanying this paper visit https://doi.org/10.1192/j.eurpsy.2022.2314. click here to view supplementary material

## References

[1] Kieling C, Baker-Henningham H, Belfer M, Conti G, Ertem I, Omigbodun O, et al. Child and adolescent mental health worldwide: evidence for action. Lancet. 2011;378(9801):1515–25. doi:10.1016/s0140-6736(11)60827-1. 22008427

[2] The Lancet Psychiatry. Adolescent mental health: reasons to be cheerful. Lancet Psychiat. 2017;4(7):507. doi:10.1016/s2215-0366(17)30190-6. 28652034

[3] Yap MBH, Pilkington PD, Ryan SM, Jorm AF. Parental factors associated with depression and anxiety in young people: a systematic review and meta-analysis. J Affect Disord. 2014;156:8–23. doi:10.1016/j.jad.2013.11.00. 24308895

[4] Parker G, Tupling H, Brown LB. A parental bonding instrument. Br J Med Psychol. 1979;52(1):1–10. doi:10.1111/j.2044-8341.1979.tb02487.x.

[5] Eun JD, Paksarian D, He J-P, Merikangas KR, Daeseleire T. Parenting style and mental disorders in a nationally representative sample of US adolescents. Social Psychiatry and Psychiatric Epidemiology 2018;53(1):11–20. doi:10.1007/s00127-017-1435-4. 29110024 10.1007/s00127-017-1435-4 PMC6823599

[6] Ma L, Gao L, Chiu DT, Ding Y, Wang W, Wang Y. Depressive symptoms prevalence, associated family factors, and gender differences: a national cohort study of middle school students in China. J Affect Disord. 2020;274:545–52. doi:10.1016/j.jad.2020.05.128. 32663987

[7] Xu W, Sun H, Zhu B, Bai W, Yu X, Duan R, et al. Analysis of factors affecting the high subjective well-being of Chinese residents based on the 2014 China family panel study. Int J Environ Res Public Health. 2019;16(14):2566. doi:10.3390/ijerph16142566. PMC6678496 31323796

[8] Chen Y, Haines J, Charlton BM, VanderWeele TJ. Positive parenting improves multiple aspects of health and well-being in young adulthood. Nat Hum Behav. 2019;3(7):684–91. doi:10.1038/s41562-019-0602-x. 31061491 PMC6625866

[9] Hames JL, Hagan CR, Joiner TE. Interpersonal processes in depression. Annu Rev Clin Psychol. 2013;9:355–77. doi:10.1146/annurev-clinpsy-050212-185553. 23297787

[10] Brun L, Pansu P, Dompnier B. The role of causal attributions in determining behavioral consequences: a meta-analysis from an intrapersonal attributional perspective in achievement contexts. Psychol Bull. 2021;147(7):701–18. doi:10.1037/bul0000331. 34855428

[11] Ge X, Hou Y. Patterns of achievement attribution of Chinese adults and their sociodemographic characteristics and psychological outcomes: a large-sample longitudinal study. Personal Individ Differ. 2022;184:111230. doi:10.1016/j.paid.2021.111230.

[12] Määttä S, Nurmi JE, Stattin H. Achievement orientations, school adjustment, and well‐being: a longitudinal study. J Res Adolesc. 2007;17(4):789–812. doi:10.1111/j.1532-7795.2007.00547.x.

[13] Glasgow KL, Dornbusch SM, Troyer L, Steinberg L, Ritter PL. Parenting styles, adolescents’ attributions, and educational outcomes in nine heterogeneous high schools. Child Dev. 1997;68(3):507–29. doi:10.1111/j.1467-8624.1997.tb01955.x. 9249963

[14] Radloff LS. The CES-D scale: a self-report depression scale for research in the general population. Appl Psychol Meas. 1977;1(3):385–401. doi:10.1177/014662167700100306.

[15] Beydoun MA, Obhi HK, Weiss J, Canas JA, Beydoun HA, Evans MK, et al. Systemic inflammation is associated with depressive symptoms differentially by sex and race: a longitudinal study of urban adults. Mol Psychiatry. 2020;25(6):1286–300. doi:10.1038/s41380-019-0408-2. 31019266 PMC6813878

[16] Harshfield EL, Pennells L, Schwartz JE, Willeit P, Kaptoge S, Bell S, et al. Association between depressive symptoms and incident cardiovascular diseases. JAMA. 2020;324(23):2396–405. doi:10.1001/jama.2020.23068. 33320224 PMC7739139

[17] Karim J, Weisz R, Bibi Z, Rehman SU. Validation of the eight-item center for epidemiologic studies depression scale (CES-D) among older adults. Curr Psychol. 2015;34(4):681–92. doi:10.1007/s12144-014-9281-y.

[18] Alvarez-Galvez J, Rojas-Garcia A. Measuring the impact of multiple discrimination on depression in Europe. BMC Public Health. 2019;19(1):435. doi:10.1186/s12889-019-6714-4. 31023286 PMC6485073

[19] Little TD, Cunningham WA, Shahar G, Widaman KF. To parcel or not to parcel: exploring the question, weighing the merits. Struct Equ Model Multidiscip J. 2002;9(2):151–73. doi:10.1207/S15328007SEM0902_1.

[20] Kakemam E, Rouzbahani M, Rajabi MR, Roh YS. Psychometric testing of the Iranian version of the TeamSTEPPS teamwork perception questionnaire: a cross-cultural validation study. BMC Health Serv Res. 2021;21(1):705. doi:10.1186/s12913-021-06739-z. 34271935 PMC8285772

[21] Browne MW, Cudeck R. Alternative ways of assessing model fit. Sociol Methods Res. 1992;21(2):230–58. doi:10.1177/0049124192021002005.

[22] Bentler PM, Bonett DG. Significance tests and goodness of fit in the analysis of covariance structures. Psychol Bull. 1980;88(3):588–606. doi:10.1037/0033-2909.88.3.588.

[23] Lt H, Bentler PM. Cutoff criteria for fit indexes in covariance structure analysis: conventional criteria versus new alternatives. Struct Equ Model Multidiscip J. 1999;6(1):1–55. doi:10.1080/10705519909540118.

[24] McLeod BD, Weisz JR, Wood JJ. Examining the association between parenting and childhood depression: a meta-analysis. Clin Psychol Rev. 2007;27(8):986–1003. doi:10.1016/j.cpr.2007.03.001. 17449154

[25] Reid M, Ramey SL, Burchinal M. Dialogues with children about their families. New Dir Child Adolesc Dev. 1990;1990(48):5–28. 10.1002/cd.23219904803 2216011

[26] McFarland C, Ross M. Impact of causal attributions on affective reactions to success and failure. J Pers Soc Psychol. 1982;43(5):937–46. doi:10.1037/0022-3514.43.5.937.

[27] Weiner B. An attributional theory of achievement motivation and emotion. Psychol Rev. 1985;92(4):548–73. doi:10.1037/0033-295X.92.4.548. 3903815

[28] Khodayarifard M, Brinthaupt TM, Anshel MH. Relationships of parents’ and child’s general attributional styles to academic performance. Soc Psychol Educ. 2010;13(3):351–65. doi:10.1007/s11218-010-9114-2.

[29] Georgiou SN. Parental attributions as predictors of involvement and influences on child achievement. Br J Educ Psychol. 1999;69(Pt 3):409–29. doi:10.1348/000709999157806. 10549242

[30] Aunola K, Stattin H, Nurmi J-E. Parenting styles and adolescents’ achievement strategies. J Adolesc. 2000;23(2):205–22. doi:10.1006/jado.2000.0308. 10831143

[31] Phillipson S. Cultural variability in parent and child achievement attributions: a study from Hong Kong. Educ Psychol. 2006;26(5):625–42. doi:10.1080/01443410500390772.

[32] Ho IT, Salili F, Biggs JB, Kit‐Tai H. The relationship among causal attributions, learning strategies and level of achievement: Hong Kong Chinese study. Asia Pac J Educ. 1999;19(1):45–58. doi:10.1080/0218879990190105.

