# Supplementary material for: The role of achievement attribution in the associations between parent–child communication and psychological well-being among adolescents: A mediation analysis
Source: Eur Psychiatry. 2022 Aug 31;65(1):e52. doi: 10.1192/j.eurpsy.2022.2314 (PMC9491076; doi:10.1192/j.eurpsy.2022.2314)
Supplement: Supplementary file 1 [file S0924933822023148sup001.docx]

**S1 Text**

**Parcels of CES-D8 scale**

**dp1:**

I was happy

I enjoyed life

**dp2:**

I felt depressed

I felt that everything I did was an effort

My sleep was restless

I felt lonely

I felt sad

I could not get going

**Goodness of fit:**

χ²/df = 5.802;

CFI = 0.990 (Comparative Fit Index);

TLI = 0.980 (Tucker-Lewis Index; Non-Normed Fit Index, NNFI);

AGFI = 0.984 (Adjusted Goodness-of-Fit Index);

RMSEA = 0.042, 90% CI [0.032, 0.052] (Root Mean Square Error of Approximation)

**Parcels of parenting bonding instrument**

**pb1:**

Parents/guardians encouraged you to do things with great effort

When you did something wrong, parents/guardians asked for reasons and guided you

**pb2:**

Parents/guardians were gentile while talking with you.

Parents/guardians encouraged you to think independently.

**pb3:**

Parents/ guardians told you reasons when asking you to do something.

Parents/guardians liked to talk with you.

Parents/guardians praised you.

**Goodness of fit:**

χ²/df = 3.796;

CFI = 0.989 (Comparative Fit Index);

TLI = 0.983 (Tucker-Lewis Index; Non-Normed Fit Index, NNFI);

AGFI = 0.987 (Adjusted Goodness-of-Fit Index);

RMSEA = 0.032, 90% CI [0.024, 0.040] (Root Mean Square Error of Approximation).

**S1 Table**

**Mediation analysis of achievement attribution tendency in the associations between parent-child communication and adolescents’ psychological well-being including potential demographic variables.**

|  | **Estimate** | **S.E.** | **p** |  | **LLCI** | **ULCI** | **Beta** |
| --- | --- | --- | --- | --- | --- | --- | --- |
| **Latent Variables:** |  |  |  |  |  |  |  |
| IA =~ ia1 | 1.000 | (0.000) | < 0.001 | *** | 1.000 | 1.000 | 0.671 |
| IA =~ ia2 | 0.913 | (0.115) | < 0.001 | *** | 0.713 | 1.181 | 0.677 |
| PBI =~ pbi1 | 1.000 | (0.000) | < 0.001 | *** | 1.000 | 1.000 | 0.734 |
| PBI =~ pbi2 | 0.932 | (0.033) | < 0.001 | *** | 0.866 | 0.995 | 0.761 |
| PBI =~ pbi3 | 0.936 | (0.031) | < 0.001 | *** | 0.877 | 0.998 | 0.798 |
| CES-D8 =~ dp1 | 1.000 | (0.000) | < 0.001 | *** | 1.000 | 1.000 | 0.549 |
| CES-D8=~ dp1 | 0.516 | (0.057) | < 0.001 | *** | 0.407 | 0.634 | 0.412 |
| **Regression Paths:** |  |  |  |  |  |  |  |
| SIP <- IA | 0.691 | (0.103) | < 0.001 | *** | 0.482 | 0.884 | 0.239 |
| SIP <- PBI | 0.398 | (0.078) | < 0.001 | *** | 0.245 | 0.557 | 0.134 |
| SIP <- eduyc1 | –0.482 | (0.196) | 0.014 | * | –0.870 | –0.112 | –0.122 |
| SIP <- eduyc2 | –0.616 | (0.198) | 0.002 | ** | –1.049 | –0.241 | –0.155 |
| SIP <- eduyc3 | –0.525 | (0.193) | 0.007 | ** | –0.904 | –0.155 | –0.113 |
| SWB <- IA | 0.598 | (0.114) | < 0.001 | *** | 0.385 | 0.840 | 0.208 |
| SWB <- PBI | 1.027 | (0.081) | < 0.001 | *** | 0.865 | 1.203 | 0.348 |
| SWB <- eduyc1 | –0.368 | (0.182) | 0.043 | * | –0.717 | –0.023 | –0.094 |
| SWB <- eduyc2 | –0.590 | (0.184) | 0.001 | ** | –0.938 | –0.216 | –0.150 |
| SWB <- eduyc3 | –0.836 | (0.194) | < 0.001 | *** | –1.190 | –0.448 | –0.182 |
| SWB <- gender | –0.275 | (0.067) | < 0.001 | *** | –0.398 | –0.134 | –0.073 |
| SWB <- agef | 0.186 | (0.080) | 0.020 | * | 0.022 | 0.340 | 0.048 |
| CES-D8 <- IA | –0.073 | (0.029) | 0.012 | * | –0.134 | –0.020 | –0.128 |
| CES-D8 <- PBI | –0.323 | (0.028) | < 0.001 | *** | –0.380 | –0.271 | –0.552 |
| CES-D8 <- eduym1 | –0.046 | (0.040) | 0.244 |  | –0.121 | 0.032 | –0.049 |
| CES-D8 <- eduym2 | –0.055 | (0.032) | 0.087 | . | –0.114 | 0.008 | –0.072 |
| CES-D8 <- eduym3 | –0.115 | (0.043) | 0.007 | ** | –0.206 | –0.036 | –0.119 |
| IA <- PBI | 0.246 | (0.041) | <0 .001 | *** | 0.167 | 0.326 | 0.239 |
| IA <- eduyc11 | –0.141 | (0.095) | 0.137 |  | –0.328 | 0.046 | –0.103 |
| IA <- eduyc12 | –0.246 | (0.096) | 0.010 | * | –0.414 | –0.048 | –0.180 |
| IA <- eduyc13 | –0.235 | (0.100) | 0.019 | * | –0.428 | –0.028 | –0.147 |
| PBI <- eduym1 | 0.047 | (0.047) | 0.322 |  | –0.042 | 0.140 | 0.029 |
| PBI <- eduym2 | 0.204 | (0.042) | < 0.001 | *** | 0.120 | 0.284 | 0.158 |
| PBI <- eduym13 | 0.315 | (0.050) | <0 .001 | *** | 0.223 | 0.412 | 0.190 |
| Defined Effects: |  |  |  |  |  |  |  |
| **Depression symptom** |  |  |  |  |  |  |  |
| Indirect effect | –0.018 | (0.007) | 0.008 | ** | –0.031 | –0.005 | –0.031 |
| Total effect | –0.341 | (0.027) | < 0.001 | *** | –0.395 | –0.287 | –0.583 |
| **Subjective well-being** |  |  |  |  |  |  |  |
| Indirect effect | 0.147 | (0.031) | < 0.001 | *** | 0.090 | 0.211 | 0.050 |
| Total effect | 1.174 | (0.078) | <0.001 | *** | 1.026 | 1.335 | 0.398 |
| **Subjective interpersonal** |  |  |  |  |  |  |  |
| Indirect effect | 0.170 | (0.034) | < 0.001 | *** | 0.103 | 0.237 | 0.057 |
| Total effect | 0.568 | (0.074) | < 0.001 | *** | 0.429 | 0.716 | 0.191 |

Note: * *p* < 0.05. ** *p* < 0.01. *** *p* < 0.001. Beta: standardized estimates.

Demographic variables including gender of adolescents, education level of adolescents and education level of mother and age of father were included as control factors to subjective well-being, subjective interpersonal popularity, depression symptom, internal attribution and parent-child communication respectively. Specific regression coefficients, indirect and total effects are shown in table.

Abbreviation: PBI, parenting bonding instrument; IA, internal attribution; CES-D8, Center for Epidemiologic Studies Depression scale; SWB, subjective well-being; SIP, subjective interpersonal popularity; eduyc1/2/3: education level of child(ref.=0); eduym1/2/3: education level of mother (ref.=0); agef: age level of father (ref.=0(28-40 yesrs)).

**Goodness of fit:**

χ²/df = 1.585

CFI = 0.989 (Comparative Fit Index)

TLI = 0.984 (Tucker-Lewis Index; Non-Normed Fit Index, NNFI)

AGFI = 0.975 (Adjusted Goodness-of-Fit Index)

RMSEA = 0.017, 90% CI [0.011, 0.022] (Root Mean Square Error of Approximation)
